# Correction: Wang et al. Decellularized Antler Cancellous Bone Matrix Material Can Serve as Potential Bone Tissue Scaffold. *Biomolecules* 2024, *14*, 907

**DOI:** 10.3390/biom16050659

**Published:** 2026-04-29

**Authors:** Yusu Wang, Ying Zong, Weijia Chen, Naichao Diao, Quanmin Zhao, Chunyi Li, Boyin Jia, Miao Zhang, Jianming Li, Yan Zhao, Rui Du, Zhongmei He

**Affiliations:** 1 College of Chinese Medicinal Materials, Jilin Agricultural University, Changchun 130118, China; wangyusu@mails.jlau.edu.cn (Y.W.); zongying@jlau.edu.cn (Y.Z.); weijiac@jlau.edu.cn (W.C.); diaonaichao@jlau.edu.cn (N.D.); zhaoquanmin@jlau.edu.cn (Q.Z.); lichunyi@cstu.edu.cn (C.L.); zhangmiao96@mails.jlau.edu.cn (M.Z.); lijianming773@jlau.edu.cn (J.L.); zhaoyan@jlau.edu.cn (Y.Z.); 2 Institute of Antler Science and Product Technology, Changchun Sci-Tech University, Changchun 130112, China; 3 College of Animal Medicine, College of Animal Science and Technology, Jilin Agricultural University, Changchun 130118, China; jiaboyin@jlau.edu.cn

## Error in Table 3

In the original publication [1], a data entry error occurred in Table 3: the standard deviation for element C was inadvertently copied from that of element O. Upon verification against the original data, it was confirmed that the data for elements C and O are not within the scope of this study and were redundant information introduced during table preparation. Therefore, this correction is made as follows: the rows for elements C and O have been removed from Table 3.

**Table 3:** Energy-dispersive X-ray spectroscopy (EDS).

Elements	Chemical Compositions (wt%)			
	NACB	DACB	XKC	TCP
P	13.73 ± 3.57	11.20 ± 2.42	15.41 ± 1.22	19.27 ± 2.18
Ca	27.65 ± 6.69	20.10 ± 5.28	30.25 ± 8.45	36.62 ± 4.34
Mg	0.45 ± 0.13	0.58 ± 0.21	——	——
Ca/P	2.02	1.78	1.96	1.90

This correction does not affect the conclusions of the paper in any way. The primary elements of interest in this study are calcium (Ca), phosphorus (P), and magnesium (Mg), whereas carbon (C), oxygen (O), and sodium (Na) are presented solely as background compositional elements. Their removal does not affect any core comparisons or the derivation of the study’s conclusions. Accordingly, this correction does not alter any of the findings. The authors state that the scientific conclusions are unaffected. This correction was approved by the Academic Editor. The original publication has also been updated.
