# Supplementary material for: Decellularized Antler Cancellous Bone Matrix Material Can Serve as Potential Bone Tissue Scaffold
Source: Biomolecules. 2024 Jul 25;14(8):907. doi: 10.3390/biom14080907 (PMC11353137; doi:10.3390/biom14080907)
Supplement: Supplementary file 1 [file biomolecules-14-00907-s001.zip › biomolecules-3092765-supplementary.pdf]

Supplementary figures

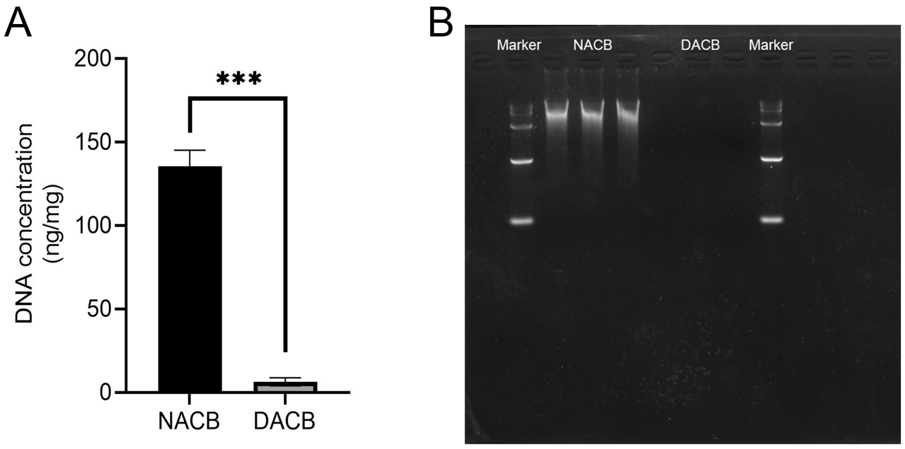

Figure S1. DNA quantification in ng/mg of NACB and DACB (A), with gel electrophoresis to detect residual DNA before and after decellularization (B). (***, P < 0.001)

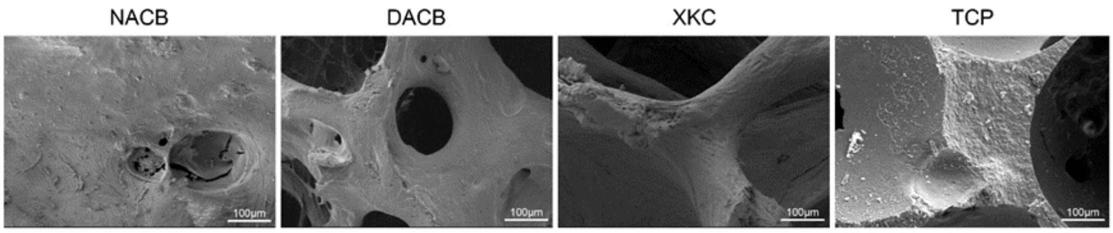

Figure S2. SEM of different materials.

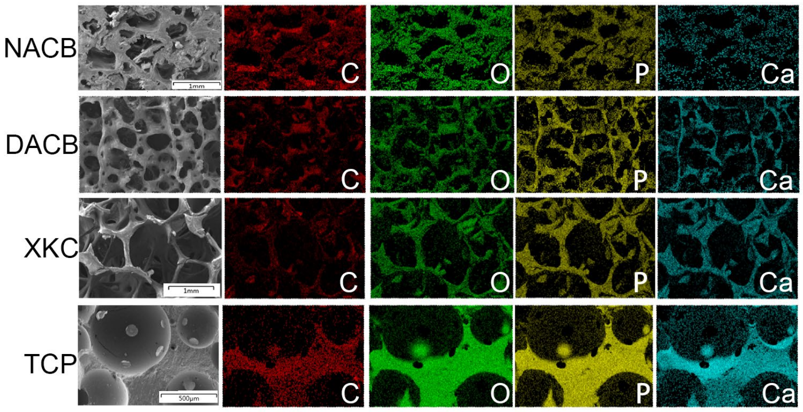

Figure S3. Microstructure of sample and EDS surface distribution of each element.

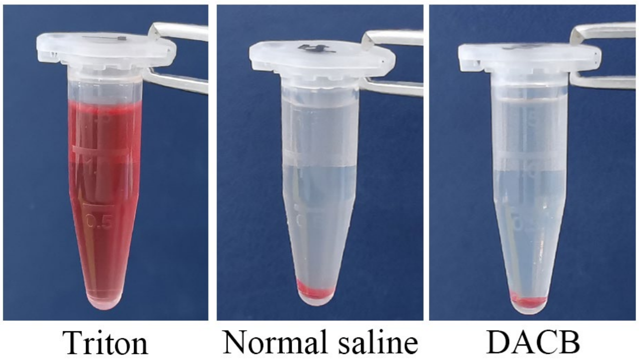

Figure S4. Hemolysis rate of DACB.

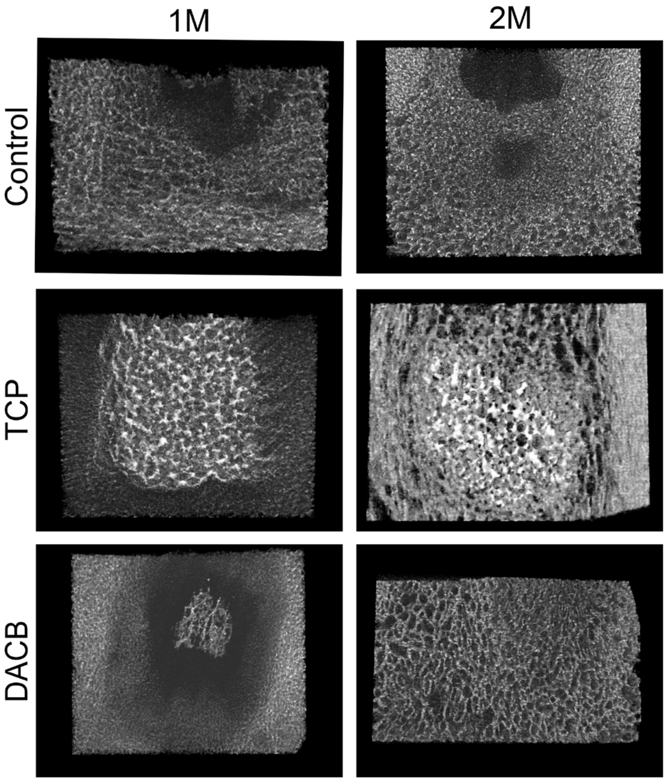

Figure S5. The transverse sections of 3D reconstructed micro-CT scanning are further used to evaluate the effect of implantation on trabecular bone adjacent to the implants.

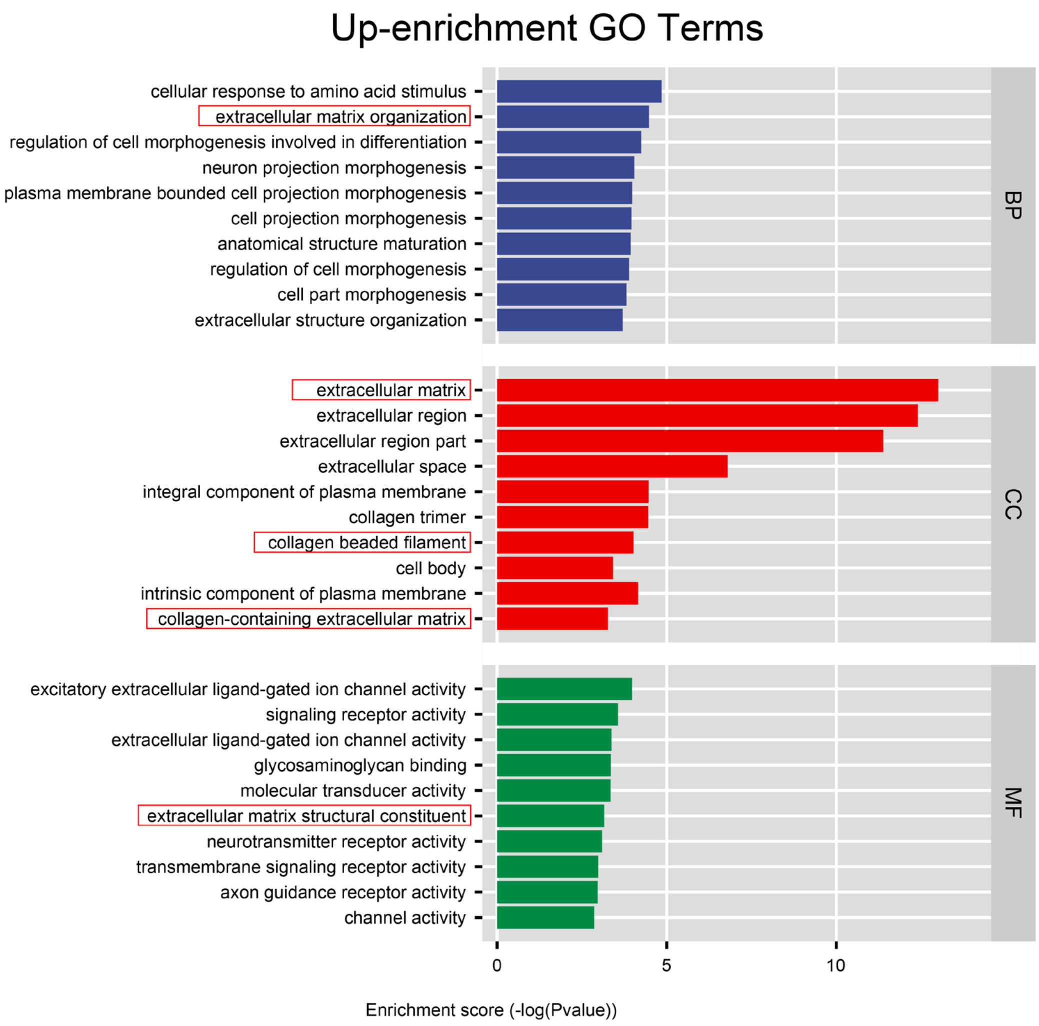

Figure S6. GO enrichment analysis of the up-regulated DEGs in DACB group compared to control group.

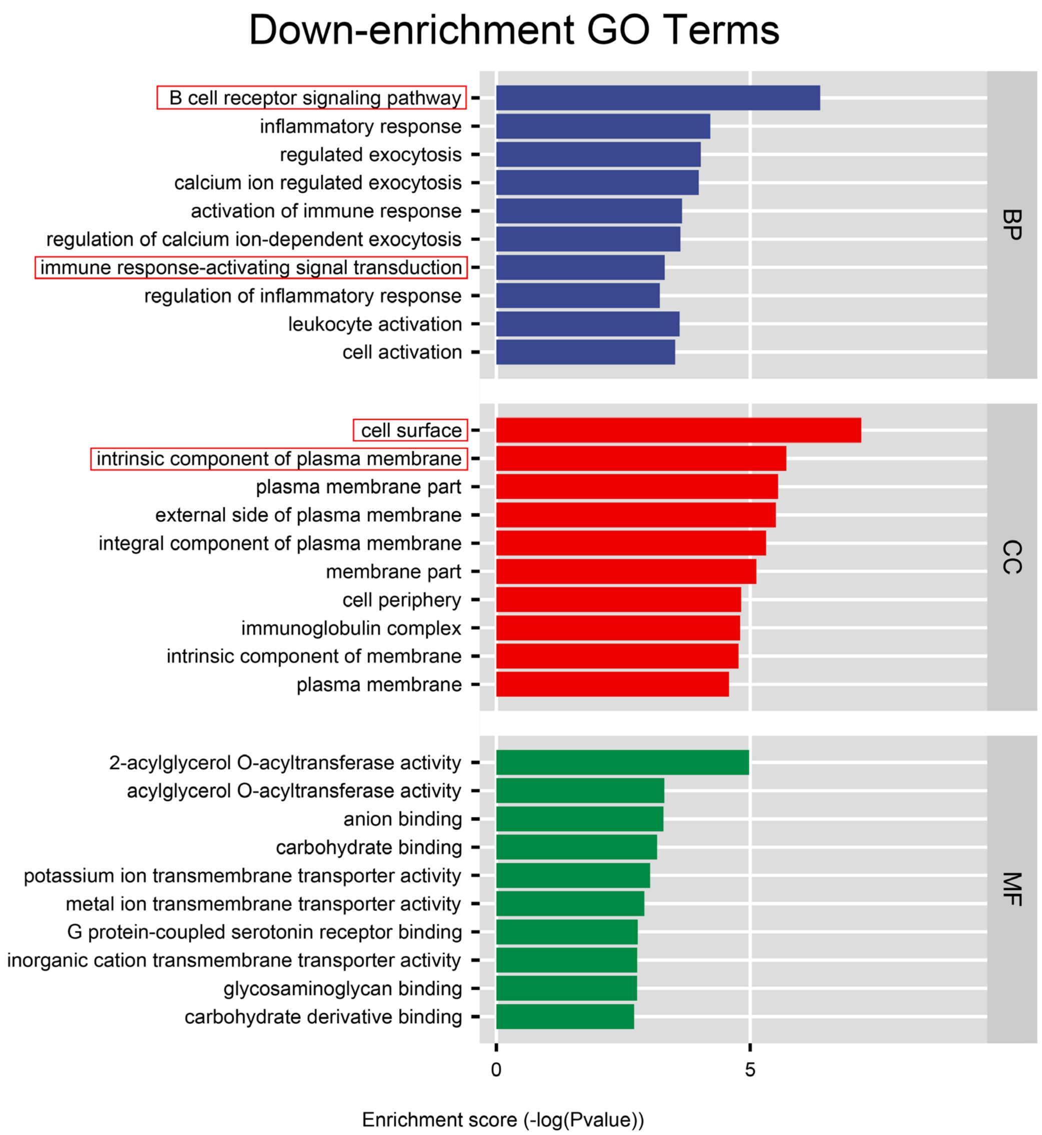

Figure S7. GO enrichment analysis of the down-regulated DEGs in DACB group compared to control group.

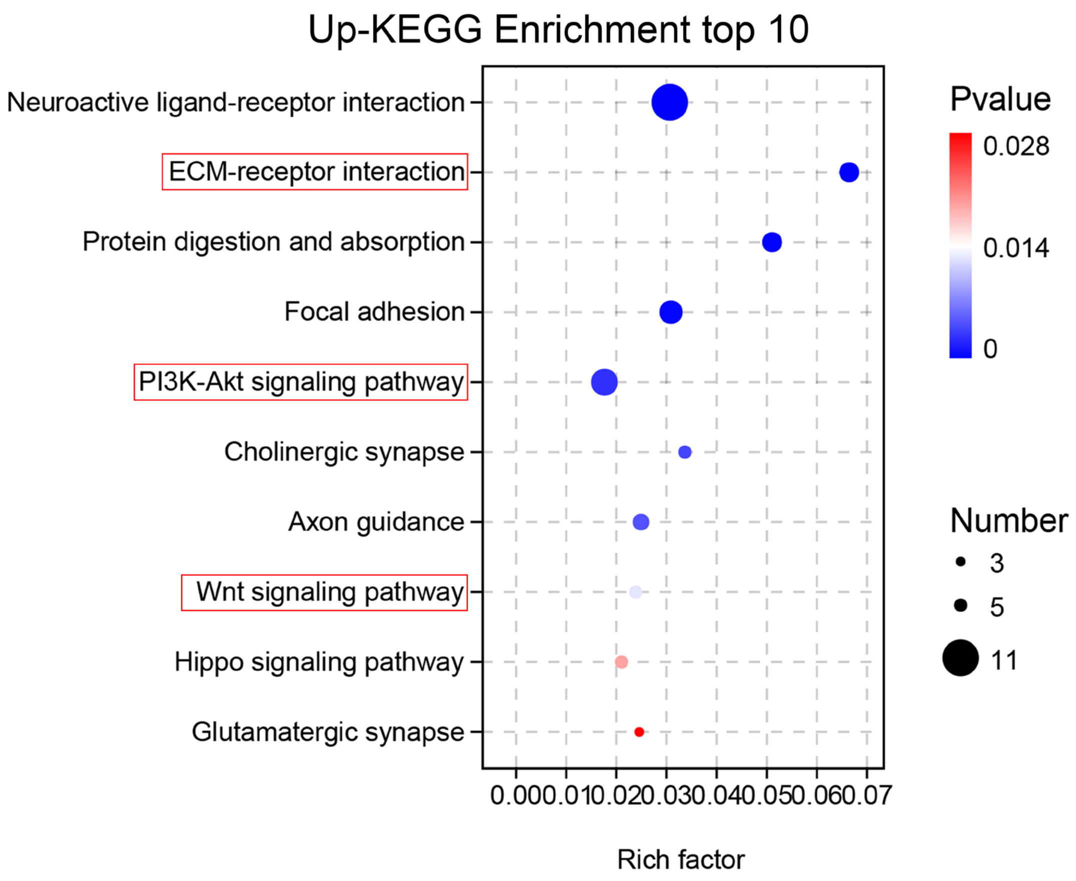

Figure S8. KEGG pathway analysis of the up-regulated DEGs in DACB group compared to control group.

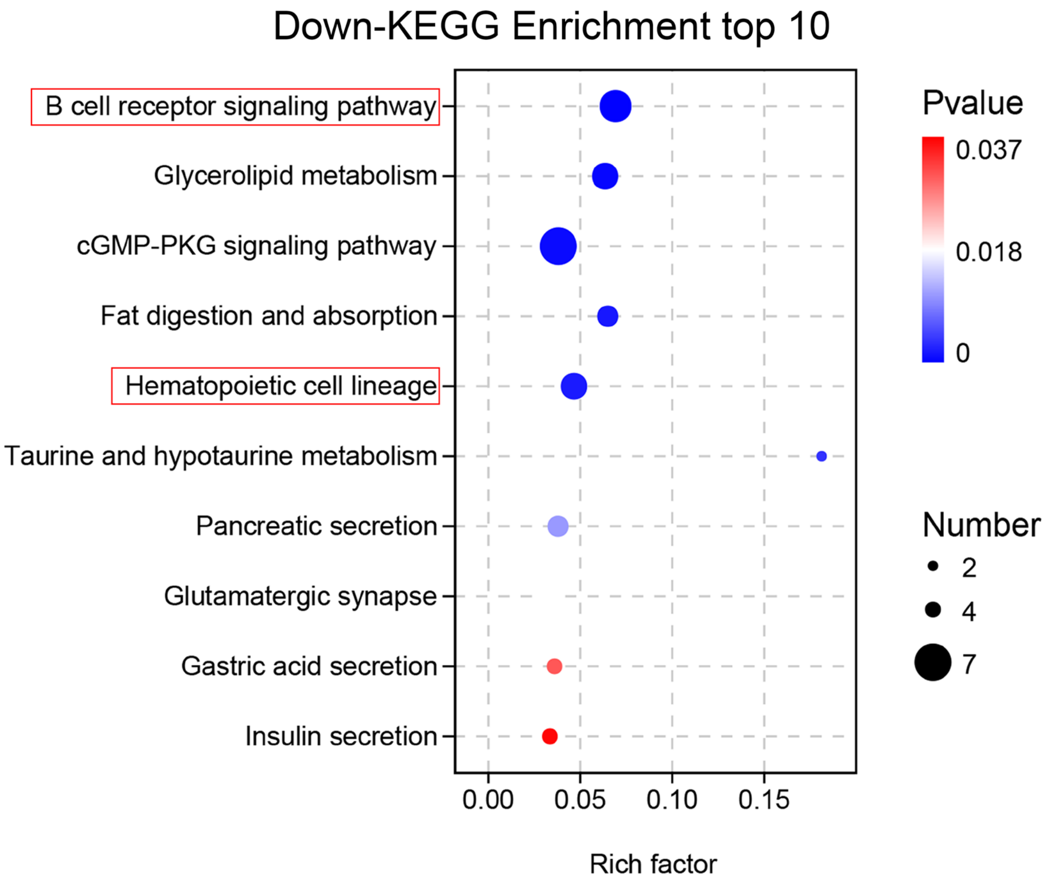

Figure S9. KEGG pathway analysis of the down-regulated DEGs in DACB group compared to control group.
