# Supplementary figures and images for: Decellularized Antler Cancellous Bone Matrix Material Can Serve as Potential Bone Tissue Scaffold
Source: Biomolecules. 2024 Jul 25;14(8):907. doi: 10.3390/biom14080907 (PMC11353137; doi:10.3390/biom14080907)

Marker

NACB

DACB

Marker

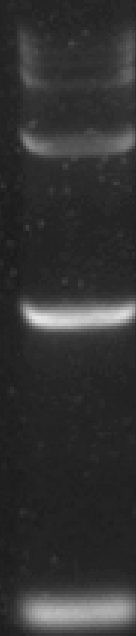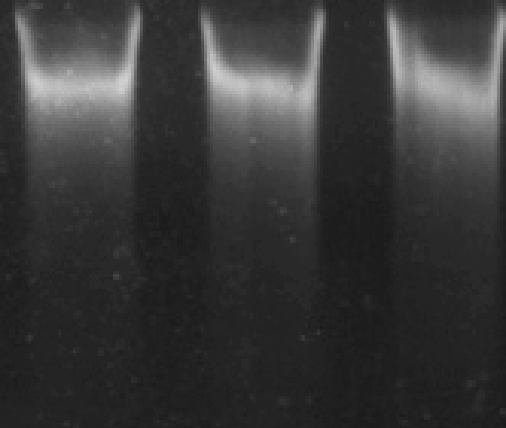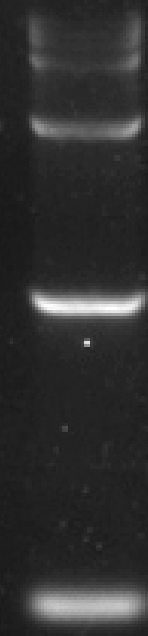

Supplement: Supplementary file 1 [file biomolecules-14-00907-s001.zip › biomolecules-3092765-original-images.pdf]
